# Dietary Patterns, Eating Disorder Risk, Emotional Distress, and Amino Acid Intake in Adults Seeking Weight Loss

**DOI:** 10.3390/nu18152497

**Published:** 2026-08-02

**Authors:** Alessia Placanica, Carmelo Pujia, Rossella Bruno, Rosy Conforto, Giovanni Luigi Tripepi, Elisa Mazza, Yvelise Ferro, Alberto Castagna, Samantha Maurotti, Arturo Pujia, Tiziana Montalcini

**Affiliations:** 1 Department of Clinical and Experimental Medicine, University “Magna Græcia” of Catanzaro, 88100 Catanzaro, Italy; alessia.placanica@studenti.unicz.it (A.P.); elisamazza@unicz.it (E.M.); smaurotti@unicz.it (S.M.); tmontalcini@unicz.it (T.M.); 2 Clinical Nutrition Unit, Renato Dulbecco Hospital, 88100 Catanzaro, Italy; carmelopujia97@gmail.com (C.P.); rosyconforto@outlook.com (R.C.); 3 Department of Medical and Surgical Sciences, University “Magna Græcia” of Catanzaro, 88100 Catanzaro, Italy; rossella.bruno001@studenti.unicz.it (R.B.); pujia@unicz.it (A.P.); 4 Institute of Clinical Physiology of the National Research Council (IFC-CNR), 56124 Reggio Calabria, Italy; 5 Research Center for the Prevention and Treatment of Metabolic Diseases, University “Magna Græcia” of Catanzaro, 88100 Catanzaro, Italy

**Keywords:** dietary patterns, eating disorder risk, distortion body image, Mediterranean diet, amino acid intake

## Abstract

**Background**: Diet quality and amino acid intake influence both physical health and psychological well-being, potentially affecting eating disorder risk. This study aimed to investigate the relationship between dietary patterns, amino acid intake, and eating disorder risk. **Methods**: This cross-sectional study included 130 adults with overweight or obesity. All participants were assessed for eating disorder risk and body image distortion. Dietary intake was assessed using a validated food frequency questionnaire. Principal Component Analysis was performed to identify major dietary patterns, and logistic regression was used to estimate the odds of eating disorder risk in relation to dietary pattern adherence and specific amino acid intake. **Results**: Six major dietary patterns were identified; among these, the mixed Mediterranean-like and Comfort Food patterns showed significant associations with eating disorder risk. Greater adherence to the mixed Mediterranean-like pattern was associated with lower odds of eating disorder risk (OR = 0.60; 95% CI: 0.39–0.93; *p* = 0.023). Conversely, adherence to the Comfort Food pattern, characterized by sweets, milk, and dairy products, showed a trend toward higher odds of eating disorder risk (OR = 1.49; 95% CI: 0.99–2.23; *p* = 0.05) and body image distortion (OR = 1.70; 95% CI: 1.03–2.79; *p* = 0.03). Regarding amino acid intake, higher consumption of glutamic acid and glycine was associated with a lower eating disorder risk, whereas higher leucine intake was associated with higher eating disorder risk. **Conclusions**: Dietary patterns are associated with eating disorder risk and body image distortion. A mixed Mediterranean-like dietary pattern may have protective effects, whereas greater adherence to a Comfort Food pattern and higher intake of specific amino acids may increase the risk of eating disorders. However, further clinical studies are needed to confirm our findings.

## 1. Introduction

Obesity is a complex chronic disease characterized by an abnormal or excessive accumulation of fat [1], which increases the likelihood of developing various noncommunicable diseases (NCDs), including type 2 diabetes, heart disease, chronic respiratory disease and several types of cancer [1]. It represents a major global health issue and has now reached pandemic levels. Recent data indicate that more than 650 million adults worldwide live with obesity, and forecasts suggest this number could reach one billion by 2030, corresponding to nearly one-fifth of the world’s population [2]. In Italy, during 2022–2023, about 40% of adults were overweight and over 10% are obese [3]. In line with adult trends, childhood overweight and obesity in Italy affect 19% and 9.8% of children, respectively [4].

Obesity is not just a problem of excess calories, but a condition with biological, psychological, and behavioral determinants [5]. Thus, understanding the complexity of psychological and behavioral profiles in individuals with obesity is essential to guide the appropriate selection of therapeutic interventions, including psychological, behavioral, and nutritional strategies [6].

It is also essential to consider diet not merely in terms of individual nutrients, but as a dietary pattern that more accurately reflects actual eating habits and their interactions with individual behaviors. In particular, emotional eating, which refers to food intake triggered by emotional states, has been consistently associated with weight gain, overweight/obesity, and unbalanced dietary patterns (high in energy-dense foods), highlighting the importance of considering overall eating patterns rather than focusing exclusively on individual foods or components of the diet [7].

Accordingly, growing scientific interest has focused on the role of dietary patterns in the regulation of eating behavior and mental health. Dietary patterns such as the Mediterranean Diet (MD) or the DASH (Dietary Approaches to Stop Hypertension) diet, rich in fruit, vegetables, fiber, and unsaturated fatty acids, have been associated with better emotional and cognitive outcomes, while “Western” diets, characterized by high consumption of simple sugars, saturated fats, and ultra-processed foods, have been linked to impulsivity, cravings, and emotional dysregulation [8]. Paradoxically, having overweight or obesity is associated with higher eating disorder risk particularly binge eating and cyclical restriction [9].

Moreover, chronic exposure to idealized body models promoted by the media—focused on thinness and low body fat—can create discrepancies between actual and ideal body image, fostering body dissatisfaction, distorted self-perception, and eating disorder risk [10,11].

Furthermore, anxiety, depression, and stress are highly prevalent among adults with obesity [12,13]. Among psychological factors, social anxiety represents an important predisposing factor as fear of judgment, particularly appearance-related evaluation may increase vulnerability to body image concerns and eating disorder risk [14,15].

Both negative and positive emotions influence eating behavior, as emotional eating may manifest as either increased food intake in response to stress, sadness, or anxiety or decreased intake during intense emotional experiences [16,17]. This mechanism is a well-documented risk factor for the development of disordered eating behaviors and, in some cases, clinical eating disorders [18].

Despite the high prevalence of psychological distress among adults with obesity, few studies have simultaneously investigated dietary patterns, emotional distress, and eating disorder risk.

A further area of interest concerns the role of amino acids, particularly those involved as precursors or modulators of neurotransmitter synthesis, in the regulation of neurobiological systems implicated in mood, appetite, and stress response. Amino acid levels should therefore be considered with caution, since, although they can be measured in blood, they largely depend on dietary intake and on the overall composition of the diet [19].

The complexity of the relationship between amino acids and eating disorder risk is further supported by the fact that amino acids do not only serve structural functions in protein synthesis, but also participate in several metabolic and neurobiological networks. In particular, recent evidence shows that associations between amino acids and metabolic networks differ across obesity phenotypes, and that their analysis may help to better characterize the transition from metabolically healthy to metabolically altered phenotypes in the presence of obesity [20].

Amino acids may also indirectly influence brain function and, potentially, vulnerability to eating disorder risk through different mechanisms. For example, glutamate/glutamic acid and glutamine are closely linked to glutathione metabolism, one of the major endogenous antioxidant systems [21]. Glycine, cysteine, and glutamic acid combine to form glutathione [22]. Therefore, reduced concentrations of amino acids involved in these metabolic pathways may be associated with lower antioxidant capacity, with possible consequences for metabolic and neurobiological processes also implicated in the regulation of food intake.

Another relevant example concerns branched-chain amino acids, particularly leucine, isoleucine, and valine. These amino acids compete with tryptophan for transport across the blood–brain barrier, mainly through the LAT1 transporter. Since this transport system is limited and saturable, increased peripheral availability of branched-chain amino acids may reduce tryptophan entry into the central nervous system, potentially influencing brain serotonin synthesis, a neurotransmitter involved in the regulation of mood, satiety, and eating behavior [23].

Overall, the relationship between dietary amino acid intake, peripheral availability, and neurobiological function appears complex and non-linear. To date, the association between amino acids introduced through the diet and eating disorder risk, understood as a set of attitudes, emotions, and behaviours related to eating, has not been fully explored. These findings suggest that the analysis of dietary amino acids may provide relevant information to better understand the biological mechanisms linking diet and vulnerability to disordered eating behaviors.

For these reasons, the aim of this study was to investigate the association between different dietary patterns, amino acid intake, and eating disorder risk, as well as the presence of psychological symptoms (anxiety, stress, and depression) in an adult population.

## 2. Materials and Methods

### 2.1. Population

This cross-sectional study was conducted from January to October 2025. It included adults of both sexes, aged ≥18 years, who were overweight or obese and who spontaneously sought treatment for weight loss through dietary intervention at the Clinical Nutrition Unit of the “Renato Dulbecco” University Hospital in Catanzaro, Italy. Overweight was defined as a body mass index (BMI) of 25.0–29.9 kg/m^2^ and obesity as a BMI ≥ 30.0 kg/m^2^, according to World Health Organization (WHO) criteria [24]. Participants were eligible if they were free from diseases that could influence nutritional status (including cancer, coronary heart disease, and endocrine disorders) and were not taking medications known to affect energy intake or metabolism. Exclusion criteria also included inability to read or complete the study questionnaires and psychometric assessments. A history of diagnosed psychiatric disorders, including depression, was considered an exclusion criterion. However, the presence of subclinical symptoms of depression, anxiety, stress, and disordered eating was not an exclusion criterion and was assessed in the study population using validated self-report questionnaires.

### 2.2. Ethics

The study protocol was approved by the Territorial Ethics Committee of the Calabria Region (Protocol Register No. 304/2024). Written informed consent was obtained from all participants before enrollment. The study was conducted in accordance with the ethical principles of the Declaration of Helsinki [25]. All data were collected and processed in accordance with the General Data Protection Regulation (EU) 2016/679 (GDPR). Participants’ data were anonymized before analysis to ensure confidentiality.

### 2.3. Anthropometric Measurements and Body Composition Assessment

Body weight was measured with participants lightly clothed using a calibrated digital scale (Tanita BC-418MA model, Tokyo, Japan) [26], and the weight of clothing was subsequently deducted. Height was measured with the participant standing upright using a wall-mounted stadiometer (seca 213 model, Hamburg, Germany) [26]. Body Mass Index (BMI) was calculated as weight (kg)/height (m^2^). Waist circumference (WC) was measured using a flexible measuring tape. All participants also underwent bioelectrical impedance analysis (BIA; Nutrilab, Akern Srl, Florence, Italy) [27] to assess body composition. Sensor electrodes were placed at the midpoint between the distal prominences of the radius and ulna on the right wrist and between the medial and lateral malleoli of the right ankle. After approximately five minutes in the supine position, resistance (R) and reactance (Xc) were measured. BodyGram Plus software (version 3.0.33; Akern, Florence, Italy) was used to estimate fat mass (FM) and skeletal muscle mass (SMM). Measurements followed standardized protocols, and all assessments were performed by the same trained operator.

### 2.4. Biochemical Evaluation

Venous blood samples were obtained in the morning after an overnight fast and collected in vacutainer tubes (Becton & Dickinson, Plymouth, England). All samples were processed within 4 h of collection by centrifugation. Serum concentrations of glucose, creatinine, total cholesterol (TC), triglycerides (TGs), high-density lipoprotein cholesterol (HDL-C), alanine aminotransferase (ALT), aspartate aminotransferase (AST) and gamma-glutamyl transferase (GGT) were determined using chemiluminescent immunoassay methods on a COBAS 8000 analyzer (Roche International Ltd, Rotkreuz, Switzerland), following the manufacturer’s protocols [26]. Quality control assessments were performed daily for all measurements.

### 2.5. Food Groups and Nutrients Intake Assessment

Dietary intake data were collected using a food frequency questionnaire (FFQ) to assess the frequency and portion sizes of foods and beverages consumed over the past month [28]. In this study, the FFQ was administered by a registered dietitian rather than self-reported. To account for known systematic errors in FFQs and ensure internal calibration, a 24-h dietary recall (24HR) was also administered. Portion sizes were based on typical or natural units (e.g., an egg, a slice of bread) or commonly used measures (e.g., cup, tablespoon), with images provided to improve accuracy. All data were linked to the MetaDieta 3.0.1 nutrient composition database (San Benedetto del Tronto, Italy), which draws on INRAN 2000, IEO 2008 and 2015, CREA 2019, and USDA databases, including ORAC values and nutrient content. This software includes over 6500 food items and up to 150 components, updated annually, and categorizes foods into major groups such as fruits, vegetables, legumes, cereals, potatoes, milk, eggs, fish, meat, sugary beverages, cake/pies and olive oil [28].

### 2.6. Assessment of Eating Disorder Risk and Presence of Psychological Symptoms

All participants completed questionnaires assessing eating behavior, dietary habits, body dissatisfaction, reflective functioning, and symptoms of anxiety, depression, and stress. The psychological battery, administered via Google Forms (https://forms.gle/kcgJQBhqMXJa9BnY9; accessed on 16 December 2024), included validated instruments evaluating eating disorder risk, psychological symptoms, and body image concerns. The risk of eating disorders was assessed using Eating Attitudes Test-26 (EAT-26) [29], a screening instrument that helps identify individuals at risk for conditions such as Anorexia Nervosa, Bulimia Nervosa, Binge Eating Disorder (BED), and Other Specified Feeding or Eating Disorder (OSFED). In particular, although it is not diagnostic, the EAT-26 evaluates three dimensions: dieting (concerns about weight, body shape, and food), bulimia and food preoccupation (including binge eating, self-induced vomiting, and use of laxatives), and oral control (perceived social pressure and interpersonal conflicts related to food). The questionnaire consists of 26 items rated on a six-point Likert scale ranging from “always” to “never.” A total score of 20 or higher indicates an increased risk of eating disorder [29] and suggests the need for further clinical evaluation. Body image concerns were evaluated using the Body Image Scale (BIS) [30], which assesses dissatisfaction or distress related to body perception. Scores of 10 or higher indicate a clinically significant level of body image disturbances [31]. Depressive, anxiety, and stress symptoms were assessed using the Depression Anxiety Stress Scales-21 (DASS-21) [32]. The depression subscale captures dysphoria, hopelessness, devaluation of life, anhedonia, and reduced involvement. The anxiety subscale reflects autonomic arousal and situational anxiety, while the psychological stress subscale assesses persistent tension, irritability, agitation, difficulty relaxing, and hyperarousal. Participants rate each of the 21 items on a four-point scale from 0 (“did not apply”) to 3 (“applied most of the time”), and each subscale is interpreted across severity ranges from normal to extremely severe. The 21-item version (DASS-21) is an abbreviated form of the original 42-item scale. It consists of three subscales, each comprising 7 items, and the total score for each subscale ranges from 0 to 21. To allow comparison with the original 42-item version, it is standard practice to multiply each DASS-21 subscale score by 2, resulting in a score range of 0 to 42. To identify the presence of symptoms of depression, anxiety, and stress, the following cutoff scores were applied to the doubled subscale scores: Depression ≥ 10, Anxiety ≥ 8, and Stress ≥ 15 [32].

### 2.7. Sample Size

For the sample size calculation, we assumed a correlation of r = 0.25–0.30 between the risk of eating disorder (EAT-26 ≥ 20) and adherence to a dietary pattern [33]. A total of 123 participants would be required to achieve 80% power with a two-tailed alpha of 0.05.

### 2.8. Statistical Analysis

The normality of data distribution was assessed using the Kolmogorov–Smirnov test. Continuous variables are expressed as mean ± standard deviation (SD) for normally distributed data or as median and interquartile range (IQR) for non-normally distributed variables, while categorical variables are reported as percentages (%).

The internal consistency of the questionnaires was assessed using Cronbach’s alpha coefficients, which were 0.846 for EAT-26, 0.859 for BIS-10, and 0.952 for DASS-21, indicating good internal consistency in the present sample.

To identify dietary patterns within the population, a Principal Component Analysis (PCA) was performed using the daily intake of various nutrients (carbohydrates, lipids, proteins, saturated fatty acids, monounsaturated fatty acids, polyunsaturated fatty acids, cholesterol, fiber, and alcohol) and food groups (cereals, legumes, fruit, vegetables, fish, meat, eggs, milk and dairy products, olive oil, and cakes/pies). Sampling adequacy was assessed using the Kaiser–Meyer–Olkin (KMO) statistic and Bartlett’s test of sphericity. The analysis was performed on the correlation matrix of standardized variables. The number of components to retain was determined by examining the scree plot of eigenvalues and applying Kaiser’s criterion (eigenvalues > 1). To facilitate interpretation of the dietary patterns and obtain uncorrelated components, an orthogonal rotation (varimax with Kaiser normalization) was applied to improve interpretability. Dietary patterns were named according to the highest factor loadings (r > 0.5). Participants were subsequently classified into six groups based on the factor scores associated with the identified dietary patterns. A Pearson correlation analysis was conducted to examine the relationship between psycho-emotional test scores and the dietary patterns derived from the PCA. Then, logistic regression analyses were performed to assess which dietary patterns, indicated by the Pearson correlations, were associated with the risk of eating disorder and with the presence of symptoms such as anxiety, depression, and stress. Furthermore, the PCA-derived dietary patterns (i.e., mixed Mediterranean-like pattern and Comfort Food Pattern) associated with an increased risk of eating disorder risk or with the presence of anxiety, depression, or stress symptoms were further divided into tertiles of adherence. Analysis of variance (ANOVA) and χ2 tests were used to assess differences in means and prevalences, respectively, across tertiles of adherence to the dietary patterns. Differences in non-normally distributed variables (serum glucose, γGT, and creatinine) were assessed using the Kruskal–Wallis test. Adjusted psycho-emotional test scores across tertiles of adherence to dietary patterns were calculated using analysis of covariance (ANCOVA). Additionally, sensitivity analyses were performed to exclude the potential influence of metabolic disorders on dietary habits. These analyses were conducted after excluding participants with type 2 diabetes and dyslipidemia.

Moreover, a Pearson correlation analysis was subsequently performed to investigate the relationship between daily amino acid intake and the risk of eating disorder. Finally, a logistic regression analysis was conducted to identify the amino acids associated with the risk of eating disorder, as previously indicated by the Pearson correlations. Statistical significance was set at *p* < 0.05 (two-tailed). All analyses were performed using SPSS version 29.0 for Windows (S. Wacker Drive, Chicago, IL 60606, USA).

## 3. Results

Table 1 presents the demographic and clinical characteristics of the study population. Participants had a mean age of 47 ± 14 years; 31% were male, and 71% were classified as having obesity. Overall, 32% of participants were at eating disorder risk (EAT-26 ≥ 20), while 78% reported high levels of body image distortion (BIS ≥ 10).

Energy, nutrient, and food group intakes are reported in Table 2. Mean energy intake was 2120 ± 487 kcal/day. PCA identified six distinct dietary patterns, which collectively explained 67% of the variance in nutrient composition. The patterns were defined as follows: 1. Western—characterized by higher intakes of total lipids, saturated, monounsaturated, and polyunsaturated fatty acids, and proteins; 2. Mixed Mediterranean-like—characterized by the consumption of cereals, meat, fish, eggs, and olive oils; 3. Comfort Food—characterized by higher intakes of milk, dairy products, cakes, and pies; 4. Fruitarian—characterized by a high consumption of fruit; 5. Alcoholic—characterized by a higher intake of alcoholic beverages; and 6. Plant-Based—characterized by a higher consumption of vegetables.

The factor loadings obtained from the PCA conducted using dietary variables are presented in Supplemental Table S1. Data adequacy for PCA was assessed using the Kaiser–Meyer–Olkin measure of sampling adequacy (KMO = 0.56) and Bartlett’s test of sphericity, which was statistically significant, χ^2^ (*df* = 136) = 911.7, *p* < 0.001. Six dietary patterns were retained according to Kaiser’s criterion, because all retained components had eigenvalues >1, and visual inspection of the scree plot. The eigenvalues of the six retained components were 4.17, 2.21, 1.36, 1.33, 1.27, and 1.07, respectively. Collectively, these six dietary patterns explained 67.19% of the total variance. Specifically, the first pattern explained 23.15% of the variance, the second 10.85%, the third 8.96%, the fourth 8.35%, the fifth 7.97%, and the sixth 7.90%.

The correlations between dietary patterns and psycho-emotional parameters are presented in Supplemental Table S2. Eating disorder risk (EAT-26 ≥ 20) was negatively correlated with the mixed Mediterranean-like pattern and positively correlated with the Comfort Food pattern. No significant correlations were found between eating disorder risk and the remaining dietary patterns.

Body image distortion risk (BIS ≥ 10) was positively correlated with both the Western and Comfort Food patterns (Supplemental Table S2). Anxiety symptoms were negatively correlated with the mixed Mediterranean-like pattern. No additional significant associations were observed between the other dietary patterns and either eating disorder risk or psychological symptoms.

Table 3 presents the results of the logistic regression analysis assessing the associations between dietary patterns and eating disorder risk. Greater adherence to the mixed Mediterranean-like pattern was associated with significantly lower odds of eating disorder risk (B = −0.506; *p* = 0.023; OR = 0.60; 95% CI: 0.39–0.93). In contrast, the Comfort Food pattern showed a trend toward higher odds of eating disorder risk (B = 0.401; *p* = 0.05; OR = 1.49; 95% CI: 0.99–2.23).

Regarding body image distortion, a significant association was observed only for the Comfort Food pattern (B = 0.532; *p* = 0.03; OR = 1.70; 95% CI: 1.03–2.79). By contrast, anxiety was not associated with any of the identified dietary patterns.

Supplemental Table S3 presents the demographic and clinical characteristics of the study population according to tertiles of adherence to the Mixed Mediterranean-like pattern. Participants in Tertile 3, corresponding to the highest level of adherence, included higher proportions of males and smokers and had higher WHR, MM, serum creatinine, and LDL-cholesterol levels than participants in Tertile 1, corresponding to the lowest level of adherence (Supplemental Table S3). Participants in Tertile 3 also had significantly higher mean EAT-26 scores and significantly lower BIS scores than those in Tertile 1.

**Figure 1:**
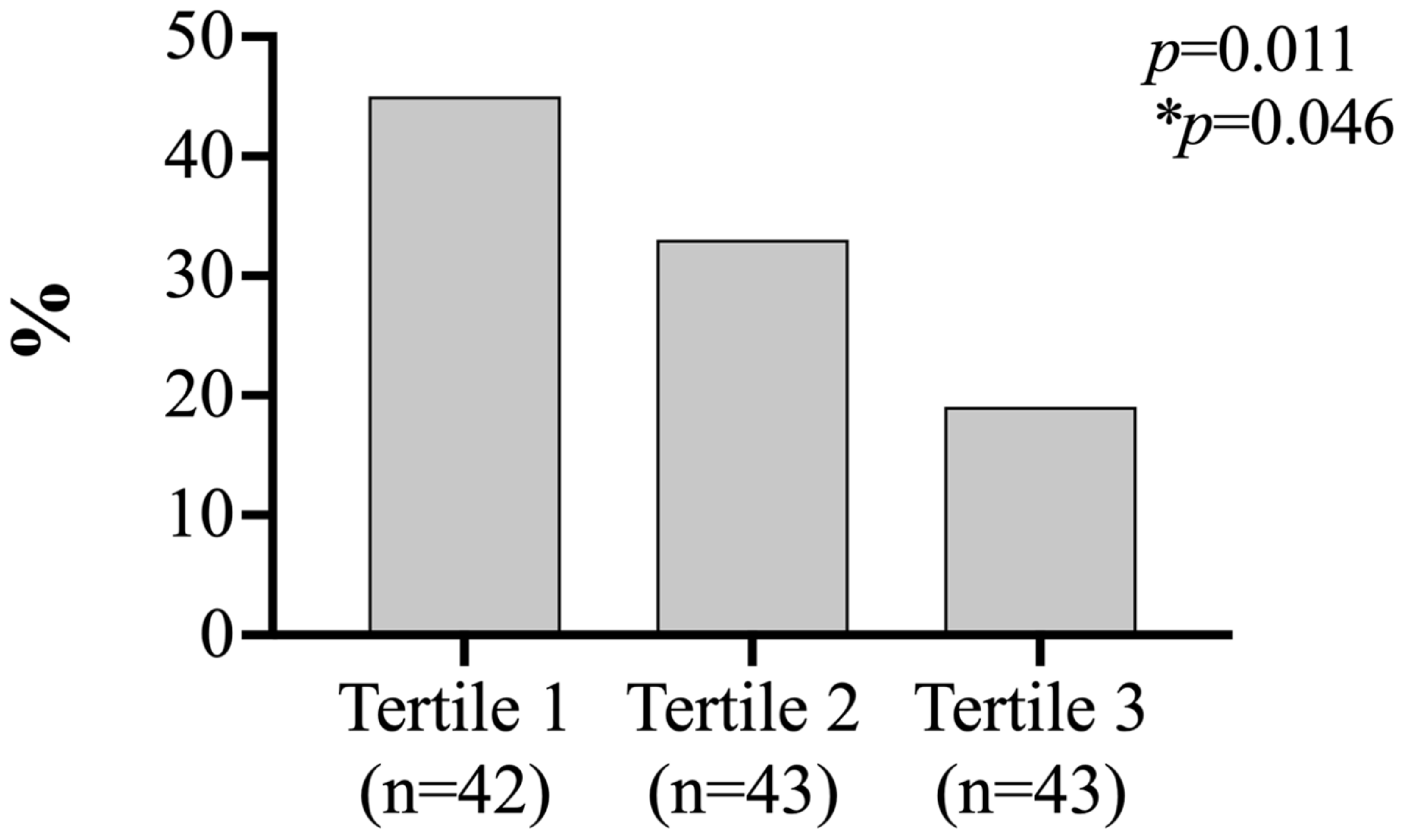
Percentage of participants classified as at risk for eating disorders according to tertiles of adherence to the mixed Mediterranean-like pattern. * Adjusted for sex, smoking status, anxiety, waist-to-hip ratio, total cholesterol and creatinine levels.

The difference in mean EAT-26 scores across tertiles remained statistically significant after adjustment for gender, smoking status, anxiety, WHR, total cholesterol, and creatinine levels (Supplemental Table S3). As shown in Figure 1, the prevalence of eating disorder risk (EAT-26 ≥ 20) decreased across increasing tertiles of adherence to the Mixed Mediterranean-like pattern (Tertile 1: 45%, Tertile 2: 33%, Tertile 3: 19%; *p* = 0.011). This association remained significant after adjustment for gender, smoking, anxiety, WHR, total cholesterol, and creatinine (Tertile 3 vs. Tertile 1; *p* = 0.046).

To exclude the potential influence of metabolic disorders on dietary habits, the analysis was repeated after participants with type 2 diabetes and dyslipidemia had been excluded. In this analysis, participants in the highest tertile of adherence to the Mixed Mediterranean-like pattern had a lower prevalence of eating disorder risk (EAT-26 ≥ 20) than those in Tertiles 1 and 2 (Tertile 3: 26% vs. Tertile 2: 36% vs. Tertile 1: 59%; *p* = 0.025).

**Figure 2:**
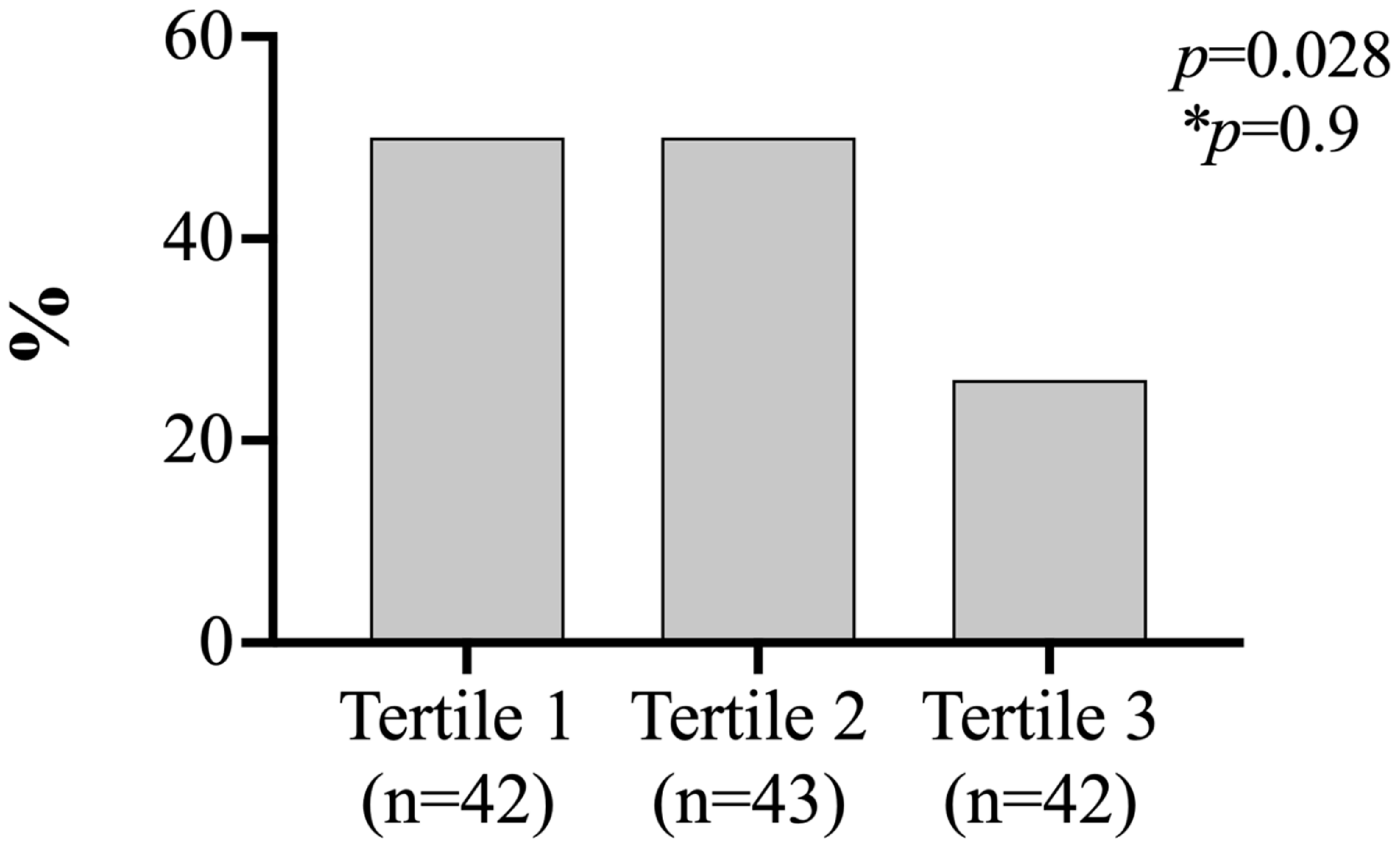
Percentage of participants with anxiety symptoms across tertiles of adherence to the mixed Mediterranean-like pattern. * Adjusted for sex, smoking status, waist-to-hip ratio, total cholesterol and creatinine levels.

Figure 2 presents the prevalence of anxiety symptoms (DASS-21 Anxiety ≥ 8) across tertiles of adherence to the Mixed Mediterranean-like pattern. Participants in Tertile 3 had a lower prevalence of anxiety than those in Tertiles 1 and 2 (Tertile 3: 26% vs. Tertile 2: 50% vs. Tertile 1: 50%; *p* = 0.028). However, this difference was no longer statistically significant after adjustment for gender, smoking, WHR, total cholesterol, and creatinine (*p* = 0.90). After excluding participants with type 2 diabetes and dyslipidemia, anxiety symptoms (DASS-21 Anxiety ≥ 8) showed a trend toward a lower prevalence in the highest tertile of adherence to the Mixed Mediterranean-like pattern (Tertile 1: 59%, Tertile 2: 52%, Tertile 3: 30%; *p* = 0.050).

Supplemental Table S4 presents the demographic and clinical characteristics of the study population according to tertiles of adherence to the Comfort Food pattern. Participants with the highest level of adherence (Tertile 3) were more frequently female and had a higher prevalence of type 2 diabetes, greater use of oral hypoglycemic agents, and higher blood triglyceride levels than participants with the lowest level of adherence (Tertile 1) (Supplemental Table S4). Participants in Tertile 3 also had significantly higher mean BIS scores than those in Tertile 1. This difference remained statistically significant after adjustment for sex, age, use of oral hypoglycemic agents, and triglyceride levels (*p* = 0.045).

**Figure 3:**
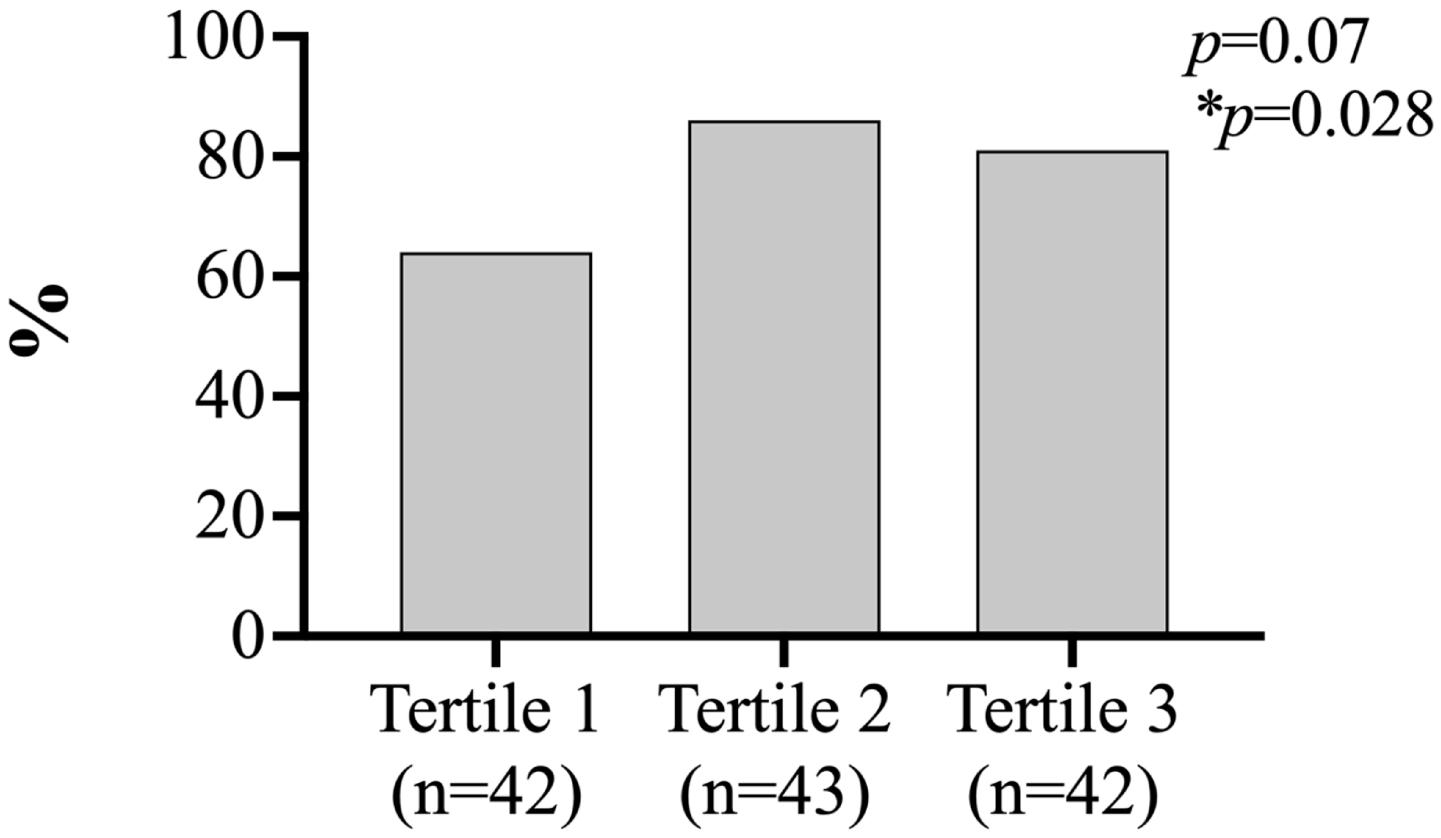
Percentage of participants at risk of body image distortion across tertiles of adherence to the Comfort Food pattern. * Adjusted for age, sex, diabetes, use of oral hypoglycemic agents and triglyceride levels.

Figure 3 presents the percentage of participants at risk of body image distortion (BIS ≥ 10) across tertiles of adherence to the Comfort Food pattern. A trend toward a higher proportion of participants at risk of body image distortion was observed in the higher-adherence tertiles than in Tertile 1 (Tertile 3: 81%; Tertile 2: 86%; Tertile 1: 64%; *p* = 0.07). After adjustment for age, sex, diabetes, use of oral hypoglycemic agents, and triglyceride levels, the difference between Tertile 3 and Tertile 1 remained statistically significant (*p* = 0.028).

Supplemental Table S5 presents the relationship between daily amino acid intake and eating disorder risk. Pearson correlation analysis showed that higher dietary intakes of histidine, arginine, aspartic acid, threonine, serine, glutamic acid, proline, glycine, alanine, cystine, isoleucine, leucine, tyrosine, phenylalanine, and tryptophan were significantly associated with a lower eating disorder risk (EAT-26 ≥ 20) (Supplemental Table S5).

No significant correlations were observed between daily amino acid intake and body image distortion risk (Supplemental Table S5). Table 4 presents the associations between eating disorder risk (EAT-26 ≥ 20) and the amino acids identified through the Pearson correlation analysis. Binary logistic regression analysis showed that higher intakes of glutamic acid and glycine were associated with lower odds of eating disorder risk, whereas higher leucine intake was associated with higher odds of eating disorder risk (Table 4).

## 4. Discussion

This study examined the associations of dietary patterns and specific amino acid intakes with eating disorder risk, and emotional distress. The study population consisted of adults seeking a weight-loss program.

Using PCA, six dietary patterns were identified: Western, mixed Mediterranean-like, Comfort Food–based, Fruitarian, Alcohol-oriented, and Plant-based. Adherence to the mixed Mediterranean-like and Comfort Food–based dietary patterns was significantly associated with the eating disorder risk (EAT-26 score ≥ 20).

In particular, greater adherence to the Mixed Mediterranean-like pattern was associated with lower odds of eating disorder risk (Table 3). This pattern was characterized by higher consumption of cereals, olive oil, meat, fish, and eggs.

Importantly, the association between adherence to the mixed Mediterranean-like pattern and lower eating disorder risk remained robust in sensitivity analyses. These analyses excluded participants with type 2 diabetes and dyslipidemia. This finding suggests that the identified association was unlikely to be driven by underlying metabolic disorders. Such disorders could potentially influence dietary behaviors and psychological outcomes.

Conversely, adherence to the Comfort Food-based pattern was associated with higher odds of eating disorder risk and body image distortion (BIS score ≥ 10) (Table 3). This pattern was characterized by high intakes of milk, dairy products, and sweets. These findings are consistent with the existing literature. Dietary patterns rich in nutrient-dense foods have been associated with more favorable psychological outcomes. Such foods include vegetables, fruits, fish, and vegetable oils. These outcomes include lower levels of anxiety and depression [34], as well as improved neurocognitive functioning.

A balanced dietary pattern has also been associated with better metabolic health. It has been linked to lower levels of systemic inflammation, and improved regulation of central nervous system processes [35].

Furthermore, such dietary patterns may protect against eating disorder risk. This protective effect may occur by fostering greater awareness of food intake and promoting psychological well-being [34].

Notably, adherence to the Mediterranean diet has been associated with a reduced risk of anorexia nervosa and bulimia nervosa. Cereals and olive oil were among the dietary components most strongly associated with these outcomes [36].

The inclusion of cereals, meat, fish, and eggs helps ensure an adequate intake of essential amino acids. These include tryptophan and tyrosine, which serve as precursors of serotonin and dopamine, respectively [35,37].

Specifically, tryptophan is the precursor of serotonin, while tyrosine is the precursor of dopamine. The availability of these amino acids is crucial for mood regulation. It is also crucial for the modulation of the brain’s reward system [38].

Accordingly, adherence to a Mixed Mediterranean-like dietary pattern may be associated with a more favorable neurochemical balance. This balance may contribute to reducing emotional dysregulation. It may also reduce compulsive food-seeking behavior, or “craving,” associated with anxiety and stress [39].

In contrast, dietary patterns characterized by a high intake of highly palatable foods have been associated with increased anxiety and depressive symptoms in adults [40]. These foods include those rich in refined sugars and saturated fats. Such dietary patterns have also been associated with higher eating disorder risk. This includes binge eating and loss of control over food intake [41].

Specifically, the Comfort Food-based pattern was significantly associated with body image distortion. This pattern was characterized by the consumption of sweets, milk, and dairy products. However, only a trend toward an association with eating disorder risk was observed.

The excessive consumption of comfort foods may contribute to the development of psychological disturbances. These foods are often used as an emotional coping strategy in response to stress. The associated psychological disturbances may include body dissatisfaction and body image distortion [42].

This behavior is commonly referred to as comfort eating. It engages the brain’s reward pathways. In this way, it reinforces food-seeking behavior aimed at emotional gratification. It may also impair impulse control. In the present study, the associations between individual amino acid intake and eating disorder risk were also examined. Higher dietary intakes of glutamic acid and glycine were associated with lower odds of eating disorder risk. By contrast, greater leucine intake was associated with higher odds of eating disorder risk (Table 4). These findings are biologically plausible in light of the currently available evidence.

Glutamic acid, or glutamate, plays a central role in brain function. It is the primary excitatory neurotransmitter in the central nervous system. It is also a precursor of γ-aminobutyric acid (GABA), the main inhibitory neurotransmitter. Glutamate is involved in maintaining the excitatory–inhibitory balance. Through this function, it may contribute to the regulation of appetite, reward processing, and impulse control. All these processes are closely implicated in eating disorder risk.

In the brain, the balance between the glutamatergic and GABAergic systems is essential for regulating emotions, impulsivity, and cognitive control [43].

A higher dietary intake of glutamic acid may increase the availability of precursors for GABA synthesis. Glutamic acid is derived from protein-rich foods, vegetables, tomatoes, and mushrooms [44]. Increased precursor availability may support an appropriate inhibitory tone. It may also promote more effective emotional self-regulation.

In turn, these factors have been associated with lower eating disorder risk. They have also been associated with a lower likelihood of restrictive behaviors, impulsivity, or food-related anxiety [45,46].

Thus, higher glutamic acid intake appears to be associated with more effective neurotransmitter regulation. Consequently, it appears to be associated with lower eating disorder risk. With regard to glycine, this amino acid exerts inhibitory functions in the spinal cord and brainstem. In the brain, it also acts as a co-agonist of the *N*-methyl-D-aspartate receptor (NMDAR). Through this mechanism, it modulates glutamatergic activity [47]. Glycine has calming, anxiolytic, and mood-stabilizing effects. It is also involved in sleep regulation and protection against oxidative stress [48].

A higher dietary intake of glycine has been associated with enhanced GABAergic tone. It has also been associated with a more favorable excitatory–inhibitory balance within the central nervous system. Glycine is derived from foods such as meat, fish, legumes, gelatin, and collagen. These effects may reduce anxiety, stress, and emotional reactivity. These factors are frequently associated with eating disorder risk [49]. Consequently, higher glycine intake may support neuroinhibitory regulation. It may also reduce the emotional and anxiety-related vulnerability linked to eating disorder risk [48,50,51].

Finally, leucine belongs to the branched-chain amino acids (BCAAs). It is found in protein-rich foods such as meat, eggs, milk, and protein supplements. BCAAs compete with tryptophan for transport across the blood–brain barrier [52]. When the intake of BCAAs, including leucine, is high, less tryptophan may enter the brain. This may result in reduced serotonin synthesis [53].

Reduced serotonin availability is associated with anxiety, depression, obsessive traits, and excessive control. It is also associated with impaired appetite and mood regulation [54]. These psychological traits are frequently observed in individuals with dietary restriction or body image concerns. Therefore, a higher intake of BCAAs, including leucine, may reduce the cerebral availability of tryptophan and serotonin. This mechanism may potentially contribute to rigidity, perfectionism, and anxiety traits linked to eating disorder risk. Although these findings are particularly interesting, they should be interpreted with caution. Several methodological limitations of the present study must be considered.

In particular, glutamic acid, glycine, and leucine were significantly associated with eating disorder risk. However, the observed effect sizes were small. This suggests that these associations may have limited clinical relevance, despite being statistically significant.

In addition, dietary amino acid intake was assessed as an indicator of habitual dietary exposure. It was not assessed as a surrogate marker of circulating or cerebral amino acid availability. Dietary amino acids may contribute to the regulation of brain-related pathways. However, their circulating concentrations are also influenced by endogenous protein turnover, tissue uptake, metabolic status, and competition among large neutral amino acids. The same factors also influence their transport across the blood–brain barrier.

Moreover, several conditions commonly associated with obesity may influence amino acid homeostasis independently of dietary intake. These include insulin resistance, chronic low-grade inflammation, and metabolic disturbances [55,56]. Therefore, the observed associations should be interpreted as relationships between habitual dietary amino acid intake and eating disorder risk. They should not be interpreted as evidence of a direct effect of individual amino acids on brain function [23,57].

Furthermore, the cross-sectional design does not allow causal relationships between dietary intake and eating disorder risk to be established.

Additionally, the study included a clinical population of adults with overweight and obesity. This may limit the generalizability of the findings to the general population.

However, the study also has several strengths. The integrated approach combines dietary pattern analysis with the assessment of specific amino acid intake and psychological evaluation. This approach allows for a more comprehensive characterization of the participants. It also represents an original contribution to the literature. Moreover, it offers new perspectives for understanding the relationship between diet quality and eating behavior.

## 5. Conclusions

This study identified six dietary patterns in adults with overweight and obesity. The findings highlighted the potentially protective role of the Mixed Mediterranean-like pattern. This pattern was associated with lower eating disorder risk. The association remained consistent in sensitivity analyses excluding participants with type 2 diabetes and dyslipidemia. This consistency supports the robustness of the findings.

Conversely, a diet rich in dairy products and sweets was associated with higher eating disorder risk and body image distortion. This diet was represented by the Comfort Food-based pattern. These findings underscore the importance of integrated nutritional interventions. Such interventions should be capable of simultaneously promoting psychological well-being and metabolic health. The assessment of dietary adherence should therefore be included in preventive strategies for eating disorders and related chronic diseases. Education regarding high-quality dietary patterns should also be included in these strategies.

In conclusion, adherence to specific dietary patterns is associated with both physical health and psychological well-being. This association highlights the importance of healthy eating habits in the context of eating disorder risk and chronic diseases in adults. Further studies are needed to confirm these findings.

## Figures and Tables

**Table 1:** Demographic, anthropometric, and clinical characteristics of the population.

Variables	Participants (n = 130)
Age (years)	47 ± 14
BMI (Kg/m^2^)	34.3 ± 6.8
WC (cm)	105 ± 17
FM (Kg)	34.3 ± 13
MM (Kg)	51.8 ± 11
Glucose (mg/dL)	93 [85, 104]
TC (mg/dL)	189 ± 43
TG (mg/dL)	126 ± 63
HDL-C (mg/dL)	55 ± 15
Creatinine (mg/dL)	0.80 [0.70, 0.96]
AST (UI/L)	24 ± 8
ALT (UI/L)	27 ± 11
γGT (UI/L)	21 [13, 27]
**Psychoemotional Test**	
EAT-26 score	14 ± 11
Eating disorder risk (EAT-26 ≥ 20, %)	32
BIS score	15 ± 6
Body image distortion (BIS ≥ 10, %)	78
Depression score	7 ± 6
Depressive symptoms (DASS-21 Depr. ≥ 10, %)	29
Stress score	10 ± 5
Stress symptoms (DASS-21 Str. ≥ 15, %)	62
Anxiety score	8 ± 5
Anxiety symptoms (DASS-21 Anx. ≥ 8, %)	42
**Prevalence**	
Gender (Male, %)	31
Menopause (%)	48
Smokers (%)	34
Physical inactivity (%)	72
Obesity (%)	71
Hypertension (%)	33
Antihypertensive agents (%)	28
T2DM (%)	10
Oral antidiabetic agents (%)	9
Hyperlipidemia (%)	44
Lipid-lowering agents (%)	41

Data are reported as mean ± standard deviation (SD) or median and interquartile range (IQR). BMI = body mass index; WC = waist circumference; FM = fat mass; MM = muscle mass; TC = total cholesterol; TG = triglycerides; HDL-C = high-density lipoprotein cholesterol; AST = aspartate aminotransferase; ALT = alanine aminotransferase; γGT = gamma glutamyltransferase; EAT-26 = 26-item Eating Attitudes Test; BIS = Body Image Scale; DASS-21= 21-item Depression Anxiety Stress Scale; T2DM = type 2 diabetes mellitus.

**Table 2:** Assessment of participants’ nutrient intake and food groups.

Variables	Participants (n = 130)
Energy Intake (Kcal/day)	2120 ± 487
Animal proteins (g/day)	50 ± 11
Plant proteins (g/day)	30 ± 7
Animal fats (g/day)	30 ± 8
Plant fats (g/day)	67 ± 28
Saturated fatty acids (g/day)	25 ± 7
Monounsaturated fatty acids (g/day)	55 ± 21
Polyunsaturated fatty acids (g/day)	12 ± 4
Cholesterol (mg/day)	304 ± 81
Carbohydrates (g/day)	232 ± 57
Fibers (g/day)	20 ± 4
Alcohol (g/day)	7 ± 10
Histidine (mg/day)	1320 ± 783
Arginine (mg/day)	2334 ± 1330
Aspartic acid (mg/day)	3804 ± 2017
Threonine (mg/day)	1793 ± 1007
Serine (mg/day)	2094 ± 1016
Glutamic acid (mg/day)	8714 ± 4265
Proline (mg/day)	2949 ± 1607
Glycine (mg/day)	1761 ± 1032
Alanine (mg/day)	2149 ± 1237
Cystine (mg/day)	618 ± 358
Isoleucine (mg/day)	2011 ± 1056
Leucine (mg/day)	3593 ± 1828
Tyrosine (mg/day)	1623 ± 887
Phenylalanine (mg/day)	2015 ± 989
Tryptophan (mg/day)	498 ± 317
Lysine (mg/day)	3046 ± 1887
Valine (mg/day)	2347 ± 1224
Methionine (mg/day)	1098 ± 643
Folic acid (mg/day)	188 ± 102
**Food groups**	
Cereals (servings/day)	2.5 ± 1.2
Legumes (servings/day)	0.3 ± 0.2
Vegetables (servings/day)	1.6 ± 0.7
Fruit (servings/day)	1.6 ± 1
Meat, fish and eggs (servings/day)	1.9 ± 0.8
Milk and dairy products (servings/day)	1.8 ± 1
Vegetable oils (servings/day)	1.7 ± 0.8
Cakes/pies (servings/day)	1.4 ± 1.2

**Table 3:** Logistic regression analysis—dietary patterns associated with eating disorder risk and body image distortion.

**Dependent Variable** **EAT-26 ≥ 20**	**B**	**SE**	**OR**	***p*** **-Value**	**CI 95%**	
					**LL**	**UL**
Mixed Mediterranean-like pattern	−0.506	0.222	0.603	0.023	0.390	0.932
Comfort Food pattern	0.401	0.206	1.494	0.050	0.999	2.235
**Dependent Variable** BIS ≥ 10 *	**B**	**SE**	**OR**	***p*** **-Value**	**CI 95%**	
					**LL**	**UL**
Comfort Food pattern	0.532	0.254	1.70	0.036	1.035	2.799

* Excluded variables: Western pattern. EAT-26 = 26-item Eating Attitudes Test; BIS = Body Image Scale; B = unstandardized coefficients; SE = standard errors; OR = odds ratio; CI = confidence interval; LL = lower limit; UL = upper limit.

**Table 4:** Logistic regression analysis—Amino acid intake and eating disorder risk.

Dependent Variable EAT-26 ≥ 20	B	SE	OR	*p*-Value	CI 95%	
					LL	UL
Model 1						
Glutamic acid	−0.001	0.000	0.999	<0.001	0.999	1.000
Glycine	−0.002	0.001	0.998	0.022	0.997	1.000
Leucine	0.002	0.001	1.002	0.001	1.001	1.003
Model 2 *						
Glutamic acid	−0.001	0.000	0.999	<0.001	0.999	1.000
Glycine	−0.001	0.001	0.999	0.030	0.997	1.000
Leucine	0.002	0.001	1.002	<0.001	1.001	1.003

Model 1: Excluded variables: serine, proline, cystine, tyrosine, phenylalanine, tryptophan, histidine, arginine, aspartic acid, threonine, alanine, isoleucine. * Model 2: Excluded variables: sex, weight, energy intake. EAT-26 = 26-item Eating Attitudes Test; B = unstandardised coefficients; SE = standard errors; OR = odds ratio; CI = confidence interval; LL = lower limit; UL = upper limit.

## Data Availability

The datasets in the present study can be obtained from the corresponding authors upon a reasonable request.

## Supplementary Materials

The following supporting information can be downloaded at: https://www.mdpi.com/article/10.3390/nu18152497/s1. Supplemental Table S1: Factor loadings of nutrients and food groups for each dietary pattern identified using principal components analysis; Supplemental Table S2: Univariate analysis—Dietary patterns related to eating disorder risk, body image distortion and psychological symptoms; Supplemental Table S3: Demographic, anthropometric, and clinical characteristics of the population according to tertiles of adherence to the mixed Mediterranean-like pattern; Supplemental Table S4: Demographic, anthropometric, and clinical characteristics of the population according to tertiles of adherence to the Comfort Food pattern; Supplemental Table S5: Univariate analysis—Correlation between amino acid intake and eating disorder risk.

## Abbreviations

The following abbreviations are used in this manuscript:

EAT-26	26-item Eating Attitudes Test
BIS	Body Image Scale
DASS-21	21-item Depression Anxiety Stress Scales
PCA	Principal Component Analysis
BMI	Body mass index
WHR	Waist-to-hip ratio
FM	Fat mass
MM	Muscle mass
TC	Total cholesterol
TG	Triglycerides
HDL-C	High-density lipoprotein cholesterol
AST	Aspartate aminotransferase
ALT	Alanine aminotransferase
γGT	Gamma glutamyltransferase
T2DM	Type 2 diabetes mellitus
