# Supplementary material for: Dietary Patterns, Eating Disorder Risk, Emotional Distress, and Amino Acid Intake in Adults Seeking Weight Loss
Source: Nutrients. 2026 Aug 2;18(15):2497. doi: 10.3390/nu18152497 (PMC13467810; doi:10.3390/nu18152497)
Supplement: Supplementary file 1 [file nutrients-18-02497-s001.zip › nutrients-4374509-supplementary.pdf]

Supplemental Table S1. Factor loadings of nutrients and food groups for each dietary pattern identified using principal components analysis

**Variables**	**Western Pattern**	**Mixed Mediterranean-like pattern**	**Comfort Food Pattern**	**Fruitarian Pattern**	**Alcoholic Pattern**	**Plant-Based Pattern**
Total Proteins	0.61					
Total Fats	0.96					
(g/die)						
Saturated fatty acids	0.84					
Monounsaturated fatty acids	0.86					
Polyunsaturated fatty acids	0.74					
Cholesterol						
Carbohydrates						
Fibers						
Alcohol					0.81	
**Food groups**						
Cereals		0.62				
Legumes						
Vegetables						0.79
Fruit				0.66		

Meat, fish and eggs	0.65	
Milk and dairy products		0.80
Olive oil	0.72	
Cakes/pies		0.67

Supplemental Table S2. Univariate analysis—Dietary patterns related to eating disorder risk, body image distortion and psychological symptoms

**Variables**		**EAT-26 ≥ 20**	**BIS ≥ 10**	**Depression**	**Stress**	**Anxiety**
Western pattern	r	-0.126	0.14	0.025	0.095	-0.043
	p-value	0.15	0.09	0.78	0.28	0.63
Mixed Mediterranean-like pattern	r	-0.207	-0.020	-0.083	0.002	-0.148
	p-value	0.020	0.82	0.35	0.97	0.09
Comfort Food pattern	r	0.179	0.187	0.139	0.081	0.031
	p-value	0.044	0.035	0.11	0.36	0.73
Fruitarian pattern	r	0.094	-0.022	-0.042	0.018	-0.085
	p-value	0.29	0.80	0.63	0.84	0.34
Alcoholic pattern	r	0.027	0.073	-0.062	0.032	-0.036
	p-value	0.76	0.41	0.48	0.72	0.68
Plant-based pattern	r	-0.142	0.002	-0.081	-0.026	-0.004
	p-value	0.11	0.98	0.36	0.77	0.96

EAT-26 = 26-item Eating Attitudes Test; BIS = Body Image Scale; DASS-21 = 21-item Depression Anxiety Stress Scale.

Supplemental Table S3. Demographic, anthropometric, and clinical characteristics of the population according to tertiles of adherence to the mixed Mediterranean-like pattern

**Variables**	**Tertile 1 (low level) (n=42)**	**Tertile 2 (medium level) (n=43)**	**Tertile 3 (high level) (n=42)**	**p-value**	**P-post hoc analysis**
Age (years)	47±14	46±13	48±14	0.64	/
BMI (Kg/m²)	33.8±8	34.2±5	34.7±7	0.83	/
WHR	0.88±0.10	0.88±0.09	0.94±0.10	0.008	1 vs 3 0.019<br>2 vs 3 0.021
FM (Kg)	33.5±14	34.6±11	34.7±14	0.89	/
MM (Kg)	47.1±10	51.1±9	57.7±12	<0.001	1 vs 3 <0.001<br>2 vs 3 0.012
Glucose (mg/dL)	90 [85-100]	91 [82-98]	95 [82-98]	0.34	/
TC (mg/dL)	176±39	197±48	195±40	0.045	1 vs 3 0.045
TG (mg/dL)	113±53	130±71	137±65	0.22	/
HDL-C (mg/dL)	56±14	56±17	52±14	0.28	/
Creatinine (mg/dL)	0.74 [0.64-0.89]	0.72 [0.68-0.89]	0.89 [0.74-1.0]	0.013	1 vs 3 0.013<br>2 vs 3 0.010
AST (UI/L)	23±8	24±8	26±7	0.27	/
ALT (UI/L)	23±11	29±12	28±10	0.08	/
γGT (UI/L)	17 [12-30]	21 [16-26]	26 [13-44]	0.32	/
**Psychoemotional tests**					
EAT-26 score	17±11	15±12	10±8	0.010	1 vs 3 0.010
*EAT-26 score	17±2	14±2	11±2	0.12	1 vs 3 0.045
BIS score	17±7	15±6	14±5	0.027	1 vs 3 0.022
*BIS score	16±1	15±1	11±1	0.12	/
Body image distortion (BIS ≥ 10, %)	79	84	69	0.30	/
Depression score	7.5±6	7.7±6	6.0±5	0.34	/
Depressive symptoms (DASS-21 Depr. ≥ 10, %)	33	33	24	0.34	/
Stress score	10±5	10±5	8±4	0.09	/
Stress symptoms (DASS-21 Str. ≥ 15, %)	62	63	57	0.65	/
Anxiety score	8±6	8±5	6±5	0.09	/

**Prevalence**					
Gender (Male, %)	14	21	57	<0.001	/
Smokers (%)	17	42	41	0.021	/
Physical activity (%)	22	26	38	0.30	/
Hypertension (%)	31	30	36	0.64	/
Antihypertensive agents (%)	29	19	36	0.44	/
T2DM (%)	14	12	5	0.15	/
Oral antidiabetic agents (%)	12	9	5	0.24	/
Hyperlipidemia (%)	48	40	45	0.82	/
Lipid-lowering agents (%)	44	40	41	0.75	/

*Adjusted for sex, smoking habit, presence of anxiety, WHR, total cholesterol and creatinine. Data are reported as mean ± standard deviation (SD) or median and interquartile range (IQR). BMI = body mass index; WHR = waist-to-hip ratio; FM = fat mass; MM = muscle mass; TC = total cholesterol; TG = triglycerides; HDL-C = high-density lipoprotein cholesterol; AST = aspartate aminotransferase; ALT = alanine aminotransferase; γGT = gamma glutamyltransferase; EAT-26 = 26-item Eating Attitudes Test; BIS = Body Image Scale; DASS-21 = 21-item Depression Anxiety Stress Scale; T2DM = type 2 diabetes mellitus.

Supplemental Table S4. Demographic, anthropometric, and clinical characteristics of the population according to tertiles of adherence to the Comfort Food pattern

Variables	Tertile 1 (low level) (n=42)	Tertile 2 (medium level) (n=43)	Tertile 3 (high level) (n=42)	p-value	P-post hoc analysis
Age (years)	43±15	50±12	47±13	0.07	/
BMI (Kg/m^2)	32.7±7	34.9±7	35.1±7	0.19	/
WHR	0.91±0.10	0.88±0.10	0.91±0.10	0.31	/
FM (Kg)	32±13	36±13	35±13	0.25	/
MM (Kg)	55±12	51±9	51±12	0.16	/
Glucose (mg/dL)	90 [83-94]	93 [86-102]	93 [83-107]	0.45	/
TC (mg/dL)	196±45	184±43	189±43	0.49	/
TG (mg/dL)	147±73	107±46	128±64	0.018	1 vs 2 0.014
HDL-C (mg/dL)	53±15	56±12	56±18	0.69	/
Creatinine (mg/dL)	0.83 [0.70-0.98]	0.80 [0.71-0.90]	0.73 [0.68-0.89]	0.21	/
AST (UI/L)	24±7	24±8	25±8	0.74	/
ALT (UI/L)	28±12	25±10	27±12	0.58	/
γGT (UI/L)	25 [12-45]	21 [13-35]	21 [13-26]	0.53	/
**Psychoemotional tests**					
EAT-26 score	14±13	13±11	16±9	0.60	/
Eating disorder risk (EAT-26 ≥ 20, %)	31	23	43	0.24	/
BIS score	14±6	15±6	17±6	0.046	1 vs 3 0.022
*BIS score	14±1	16±1	16±1	0.12	1 vs 3 0.045
Depression score	6.3±6	6.8±6	8.1±5	0.30	/
Depressive symptoms (DASS-21 Depr. ≥ 10, %)	24	28	38	0.15	/
Stress score	8.9±5	9.8±5	10.1±4	0.48	/
Stress symptoms (DASS-21 Str. ≥ 15, %)	59	60	62	0.82	/
Anxiety score	6.7±5	7.8±6	8.3±5	0.35	/
Anxiety symptoms (DASS-21 Anx. ≥ 8, %)	40	42	45	0.66	/
**Prevalence**					
Gender (Male, %)	45	27	21	0.018	/
Smokers (%)	33	30	36	0.81	/
Physical activity (%)	0	0	5	0.84	/

Hypertension (%)	27	35	33	0.64	/
Antihypertensive agents (%)	24	30	29	0.62	/
T2DM (%)	0	14	17	0.012	/
Oral antidiabetic agents (%)	0	9	17	0.07	/
Hyperlipidemia (%)	50	51	32	0.08	/
Lipid-lowering agents (%)	43	49	32	0.30	/

*Adjusted for sex, age, oral hypoglycaemic agents and triglycerides. Data are reported as mean ± standard deviation (SD) or median and interquartile range (IQR). BMI = body mass index; WHR = waist-to-hip ratio; FM = fat mass; MM = muscle mass; TC = total cholesterol; TG = triglycerides; HDL-C = high-density lipoprotein cholesterol; AST = aspartate aminotransferase; ALT = alanine aminotransferase; γGT = gamma glutamyltransferase; EAT-26 = 26-item Eating Attitudes Test; BIS = Body Image Scale; DASS-21= 21-item Depression Anxiety Stress Scale; T2DM = type 2 diabetes mellitus.

Supplemental Table S5. Univariate analysis—Correlation between amino acid intake and eating disorder risk

Variables		
		**EAT-26 ≥ 20**
Histidine	r	-0.171
	p-value	0.05
Arginine	r	-0.157
	p-value	0.07
Aspartic acid	r	-0.161
	p-value	0.06
Threonine	r	-0.154
	p-value	0.08
Serine	r	-0.201
	p-value	0.02
Glutamic acid	r	-0.267
	p-value	0.002
Proline	r	-0.245
	p-value	0.005
Glycine	r	-0.205

	p-value	0.02
Alanine	r	-0.162
	p-value	0.06
Cystine	r	-0.242
	p-value	0.006
Isoleucine	r	-0.155
	p-value	0.08
Leucine	r	-0.160
	p-value	0.07
Tyrosine	r	-0.157
	p-value	0.07
Phenylalanine	r	-0.174
	p-value	0.04
Tryptophan	r	-0.167
	p-value	0.05

EAT-26 = 26-item Eating Attitudes Test.
